# Role of SOX9 and Hif-1α expression in placentas of patients with HELLP

**DOI:** 10.1590/acb388023

**Published:** 2023-10-23

**Authors:** Senem Alkan Akalın, Ece Öcal, Engin Deveci

**Affiliations:** 1 Private Medical Practice – Department of Gynecology and Obstetrics – Bursa – Turkey.; 2 Private Medical Practice – Department of Perinatology – Diyarbakir – Turkey.; 3 Dicle University – Medical School – Department of Histology and Embryology – Diyarbakir – Turkey.

**Keywords:** Pre-Eclampsia, Placenta, Histology, Angiogenesis, Pathologic

## Abstract

**Purpose:**

In this study, we investigated the immunohistochemical staining of SRY-box transcription factor 9 (SOX9) and Hif-1α expression in placentas of pregnant woman with hemolysis, elevated liver enzymes and low platelets (HELLP) syndrome.

**Methods:**

Placentas of 20 normotensive and 20 women with HELLP syndrome were processed for routine histological tissue processing. The biochemical and clinical parameters of patients were recorded. Placentas were stained with hematoxylin-eosin and SOX9 and Hif-1α immunostaining.

**Results:**

Normotensive placentas showed normal histology of placenta, however placentas of HELLP syndrome showed intense thrombosis, thinning of the villi membrane and vascular dilatation. In placentas of normotensive patients, SOX9 reaction was immunohistochemically negative, however placentas of HELLP group showed SOX9 expression in decidual cells, and syncytial regions of floating villi and inflammatory cells. In placentas of normotensive patients, Hif-1α reaction was mainly negative in vessels and connective tissue cells. Placentas of HELLP group showed increased Hif-1α expression in decidual cell and especially inflammatory cells in the maternal region.

**Conclusions:**

Hif-1α and SOX9 proteins can be used as a marker to show severity of preeclampsia and regulation of cell proliferation and angiogenesis during placental development.

## Introduction

Many complications can develop during pregnancy, and they are called maternal morbidity. The most common diseases among pregnancy complications are gestational hypertension, preeclampsia, eclampsia, superimposed preeclampsia, gestational diabetes mellitus (GDM), postpartum hemorrhage and infections^1^,^2^. Regular pregnancy follow-ups are important for the early diagnosis of these diseases, but there are no definitive and clear screening and test according to studies. Detailed medical and obstetric history is still the most used method for diagnosis. Most pregnancy complications may be resolved after delivery, however their long-term effect may be continued future^3^,^4^.

Hemolysis, elevated liver enzymes and low platelets (HELLP) syndrome was first reported in the literature by Pritchard et al.^5^ in 1954 as severe pregnancy poisoning with hemolysis and thrombocytopenia. However, in 1982, Louis Weinstein redefined the disease as intravascular hemolysis, high liver enzymes and low platelets, which gave the disease its current name^6^. The incidence of HELLP syndrome in pregnancies is 0.2–0.76% in 1,000 live deliveries, the mortality rate is 0–24%, and the perinatal mortality rate is 37%. The incidence of HELLP syndrome is increasing in patients with eclampsia and severe preeclampsia.

**Figure 1:**
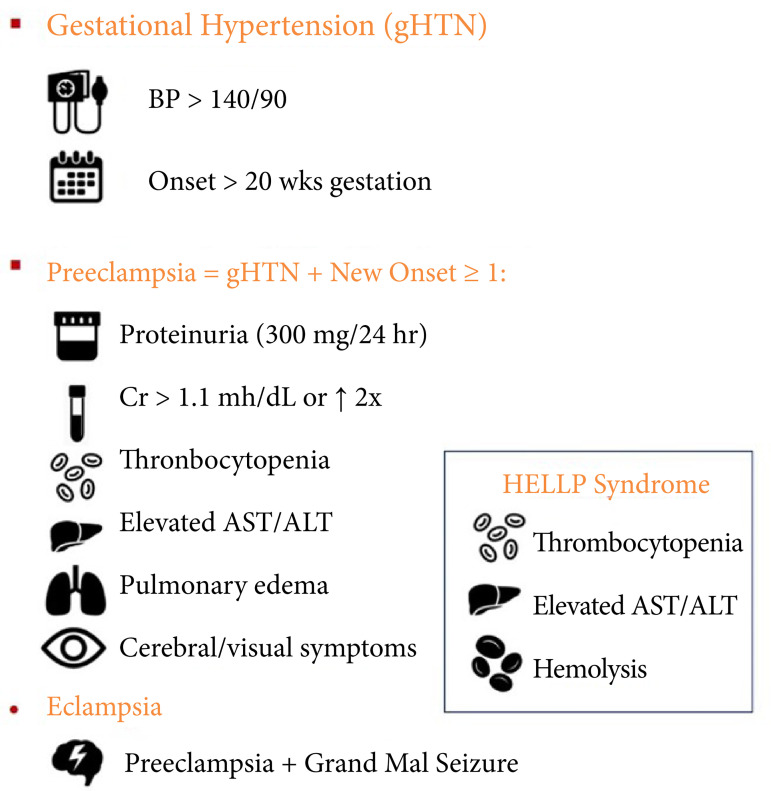
Demonstration of clinical findings of hemolysis, elevated liver enzymes and low platelets (HELLP) syndrome.

BP: blood pressure; Cr: creatinine; AST: aspartate transaminase; ALT: alanine transaminase. Source: Elaborated by the authors.

There are three different subgroups of HELLP syndrome according to the platelet count. Its clinical presentation is the same as preeclampsia, but some patients may not have hypertension or proteinuria. The pathophysiology of HELLP syndrome is still not clear nowadays. Vascular endothelial injury is the widely accepted theory. Vasoconstrictive substances are released as a result of the deterioration of vascular permeability, and it has been reported that these are found at high rates in patients with HELLP syndrome^7^,^8^ (Fig. 1). Gardikioti et al.^9^ similarly stated that pathogenesis of HELLP syndrome is linked to increase in response of maternal inflammatory and high endothelial activation. They furtherly claim that there is a relationship between ADAMTS13 and HELLP. ADAMTS13 can be used as reliable diagnostic tool for differential diagnosis of HELLP.

Transcription factors belonging to the SOX family were first identified in mammals in 1990. Y-box 9, the sex-determining region, belongs to the SOX family of transcription factors. Morphologically, it has been reported to have a vital role in survival and many developmental processes. It has also been shown that many tumors have oncogenes. It has been stated that there are important tumors that seriously threaten the life of patients in terms of gynecological malignancies. Among the most common gynecological malignancies, ovarian cancer, cervical cancer, and endometrial cancers have been reported.

Up to now, the obscurity of the molecular mechanisms related to the incidence and development of gynecological malignancies has been the main theme of the studies. Recent studies have reported that multiple mechanisms play a role in the regulation of the expression of the sex-determining region Y-box 9, which leads to the emergence and development of gynecological malignancies^10^. In addition, members of the SOX family contain a conserved high mobility group DNA binding domain.

There are 22 known SOX genes in both humans and mice, which are subdivided into nine subgroups (A, B1, B2, C, D, E, F, G, H)^11^. SOX9 is part of the SOXE subgroup, along with SOX8 and SOX10. These genes share a very conserved amino acid sequence in the HMG domain and a transactivation domain at the N-terminus. SOXE genes appear to have duplicated early during evolution since they have been detected in lampreys^12^. It appears that over time the SOXE genes have acquired other important roles in the development of many organs. SOX9 mutant mice die early during embryogenesis^13^, while SOX10 mutants show defects in neural crest differentiation^14^. SOX8 mutant mice are viable and show no detectable phenotypic abnormality, which is thought to be in part to a shared functional redundancy between all three members of the SOXE group^15^. All three members seem to have overlapping spatio-temporal expression patterns during development. For example, SOX9 is highly expressed initially during embryonic development of the pancreas, and SOX8 and SOX10 appear to be also expressed although a low levels^16^.

Hif-1 was discovered as a transcription factor for the erythropoietin protein-coding gene in humans in 1992. Studies showed that Hif-1 is a heterodimeric protein, and it consists of an Hif-1α regulatory subunit and an Hif-1β subunit. When the intracellular oxygen level decreases, prolyl hydroxylase’s enzyme activity is inhibited, and these conditions lead to nuclear transcription and synthesis of Hif-1α after the formation of the active heterodimer Hif-1β, which has a fundamental role in the transcription of hypoxia-related genes, including angiogenesis, glycolysis, hematopoiesis, and catecholamines. Nowadays, more than 2% of human genes participate in Hif-1α synthesis in arterial endothelial cells, directly and indirectly^17^, of which the vascular endothelial growth factor is the strongest endothelial mitogen^18^. Animal experiments showed that the elimination of Hif-1α gene, which encoded Hif-1α protein synthesis, stopped the progression of fetal development during days 8 to 9 and led to lethal fetal conditions during days 10 to 11, along with cardiovascular disorders and decreased hematopoiesis^19^.

The aim of this study was to investigate SOX9 and Hif-1α expression of in placentas of women with HELLP syndrome by immunohistochemical methods.

## Methods

### Patients and follow-up periods

Ethical approval was taken from Dicle University Medical Faculty Ethics Committee for Non-Interventional Clinical Studies (Date: February 28, 2023, protocol number: 74). In our study, 20 normotensive women and 20 women with HELLP syndrome were included. Placentas were obtained from gynecology and obstetrics clinics. All patients signed informed patient consent form. Biochemical and clinical parameters for each patient were recorded. Placental tissues were processed for routine paraffine wax embedding protocol.

### Histological tissue processing

Placental tissues were fixed with zinc-Formalin solution (catalog no: Z2902, Sigma-Aldrich, St. Louis, MO, United States of America) and washed under tap water by 5 minutes. Tissues were passed through ascending alcohol series for about 24 hours. Tissues were washed with xylene 2 × 30 minutes and incubated within paraffin wax. Five-μm sections were cut with microtome (catalog no: Leica RM2265, Wetzlar, Germany). Deparaffinized within xylene for 2 × 30 minutes, sections were brought to distilled water. Sections were stained with Sox-9 and Hif-1α immunohistochemical staining^20^.

### SOX9 and Hif-1α immunostaining

All placental tissues were brought to distilled water. Hydrogen peroxide solution (catalog no. TA-015-HP, ThermoFischer, Fremont, CA, United States of America) were dropped on sections for 20 minutes. After washing in phosphate buffer solution (PBS) for 3 × 5 minutes, ultra V Block (catalog no. TA-015-UB, ThermoFischer, Fremont, CA, United States of America) was applied to sections for 8 minutes. Sections were incubated with primary antibodies SOX9 and Hif-1α (catalog no. A75956 and A75872, AFG Scientific, United States of America, 1/100) at +4°C overnight. Sections were allowed to warm at room temperature for 30–60 minutes. Sections were washed with biotinylated secondary antibody (catalog no. TP-015-BN, ThermoFischer, Fremont, CA, United States of America) for 14 minutes. Streptavidin-peroxidase (catalog no. TS-015-HR, ThermoFischer, Fremont, CA, United States of America) was dropped onto sections for 15 minutes. Clearing with PBS, diaminobenzidine (DAB) (catalog no. TA-001-HCX, ThermoFischer, Fremont, CA, United States of America) was used as chromogen. Sections were counter stained with Gill hematoxylin (catalog no. 105174, Sigma-Aldrich, St. Louis, MO, United States of America) and mounted with entellan (catalog no. 107961, Sigma-Aldrich, St. Louis, MO, United States of America). Slides were analyzed with Zeiss Imager A2 Zen 3.0 software (Germany, Carl-Zeiss-Straße, Oberkochen, Germany) and photomicrographed^21^.

### Statistical analysis

The data were recorded as median (minimum – maximum). Statistical analysis was done using the IBM Statistical Package for the Social Sciences (SPSS) 25.0 software (IBM, Armonk, New York, United States of America).

## Result

### Biochemical findings

**Figure 2:**
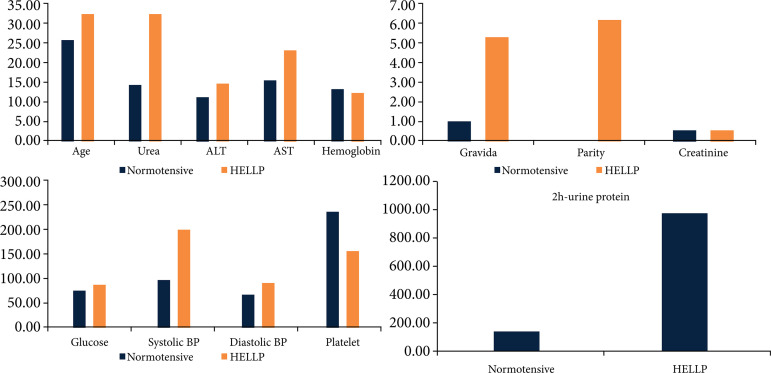
Biochemical and demographical parameters.

AST: aspartate transaminase; ALT: alanine transaminase; BP: blood pressure; HELLP: hemolysis, elevated liver enzymes and low platelets. Source: Elaborated by the authors.

Age, gravida, parity, systolic blood pressure (BP), diastolic BP, hemoglobin, platelet, glucose, urea, creatinine, alanine transaminase (ALT), aspartate transaminase (AST)-urine protein were recorded in women with normotensive and HELLP syndrome. Data are shown in Table 1. Systolic and diastolic BP level were higher, platelet level was lower in HELLP syndrome group than in normotensive group. Graphical illustration of Table 1 is shown in Fig. 2.

**Table 1:** Clinical and biochemical parameters of normotensive and hemolysis, elevated liver enzymes and low platelets (HELLP) patients.

Parameter	Normotensive (n = 20) Median (min–max)	HELLP (n = 20) Median (min–max)
Age	25 (18–33)	32 (26–40)
Gravida	1 (0–3)	5 (1–7)
Parity	0 (0–4)	6 (1–8)
Systolic blood pressure	98 (90–112)	210 (125–220)
Diastolic blood pressure	70 (60–85)	95 (87–109)
Hemoglobin	13 (10–14.5)	12.5 (9.5–13)
Platelet	248 (123–447)	163 (148–398)
Glucose	80 (68–105)	90 (72–125)
Urea	14 (12–20)	32 (18.5–48.60)
Creatinine	0.58 (0.54–0.71)	0.62 (0.53–0.84)
Alanine transaminase	11 (7–20)	14 (9–60)
Aspartate transaminase	15 (12–40)	23 (19–53)
2 h-urine protein	130 (103–180)	985 (450–1,200)

Source: Elaborated by the authors.

### SOX9 and Hif-1α findings

**Figure 3:**
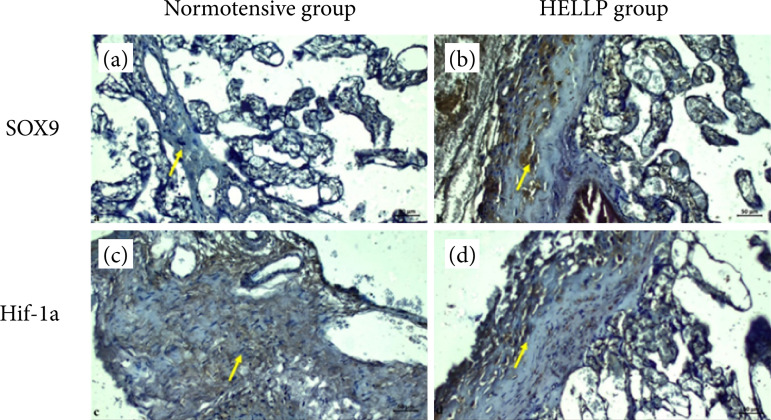
Normotensive and HELLP syndrome placentas with immune staining. Decidual cells (yellow arrows). Scale bar = 50 μm; magnification = 40x for all sections.

In the placental sections of normotensive patients, SOX9 expression was generally negative in connective tissue cells, in syncytial cells and bridges in areas with floating villi expanding with root villi. SOX9 expression was positive in some Hoffbauer cells. In addition, the SOX9 reaction was generally recorded as negative (Fig. 3a). In the placental sections of the patients with HELLP syndrome, it was clearly detected that there was enlargement of blood vessels and thrombosis with severe bleeding due to HELLP syndrome. It was observed that the decidual cells in the maternal region were hyperplastic, and the expression of SOX9 was evident. SOX9 expression was positive in the syncytial regions of floating villi and inflammatory cells, but negative in connective tissue cells (Fig. 3b).

HELLP: hemolysis, elevated liver enzymes and low platelets. Source: Elaborated by the authors.

Hif-1α reaction was evaluated as negative in the placenta sections of normotensive patients, in the areas where the umbilical cord continues and the arterial vein structure, connective tissue cells and vascular endothelium. It was evaluated that Hif-1α expression was positive in individual fibroblast cells and syncytial node regions in some collagenized areas (Fig. 3c). In the placental sections of patients with HELLP syndrome, thrombosis increased intensely, and there was thinning of the membrane structure of chorionic villi. Hif-1α expression was positive in small decidual cell nuclei and numerous clustered inflammatory cells in the maternal region. Expression of Hif-1α reaction in hyalinized areas, especially in decidual cells, was considered positive (Fig. 3d).

## Discussion

During pregnancy, complications can lead the placental abnormalities such as preeclampsia, GDM, placenta previa and accreta. HELLP syndrome have short- and long-term effects on placenta, additionally on other organs such liver and kidney. Vinnars et al.^22^ studied 196 women diagnosed with HELLP syndrome. In the histopathology of placenta, intervillous thrombosis, abruption and infarction were more common in women with HELLP syndrome. Nergiz et al.^23^ found that Hoffbauer cells were increased in placenta of HELLP syndrome. In their ultrastructural analysis, degenerative structure in cell surface membrane, intracytoplasmic edema, and degenerative vacuoles in syncytiotrophoblast were recorded. HELLP syndrome can also cause renal dysfunction due to glomerular endotheliosis and liver pathology due to apoptosis of liver sinusoidal endothelial cells, causing lesion in renal and hepatic histopathology^24^.

SRY-box transcription factor 9 or SOX9 is a transcription factor that is required for testicular development, organogenesis of liver and pancreas, cytoskeleton, and chondrocytes. Mutations in SOX9 gene can lead to autosomal sex reversal, skeletal formation, and testis development^25^,^26^. Sekido et al.^26^ studied two genes in Sertoli cell by investigating sry expression. They found that upregulation of SOX9 gene in supporting cells determine their fate as Sertoli cells, which shows importance of SOX9 gene in testis.

Zhao et al.^27^ studied endothelial to mesenchymal transition in murine endovascular progenitors. They found that endothelial to mesenchymal transition was dependent on relative expression of SOX9 along with Notch signaling, affecting their plasticity, which may be a therapy tool for fibrotic diseases. Xian et al.^28^ showed that stimulation of SOX9 can induce cellular differentiation gene, and this can be a mechanism in transformation of extra villous trophoblast to endovascular trophoblasts during placentation. In our study, normotensive placentas showed mainly negative SOX9 expression, but positive in Hoffbauer cells (Fig. 3a). In the HELLP group, SOX9 expression was intense in decidual cells, in the syncytial regions of floating villi and in inflammatory cells, but negative in connective tissue cells (Fig. 3b).

Hypoxia-inducible factor-1α is a pleiotropic transcription factor for the survival of mammalian cells under hypoxia. It affects transcription of battery of downstream genes such as erythropoietin, glucose transporters, glycolytic enzymes, vascular endothelial growth factor during hypoxic conditions. Hif-1α regulates vasculogenesis in embryonic stage, tumor angiogenesis and ischemia^29^. During placental formation, cellular hypoxia develops, thus Hif-1α is activated to induce trophoblast proliferation and the formation of specific cell subtypes^30^.

Zamudio et al.^31^ investigated role of hypoxia in placentas of women living in high (3,100 m), moderate (1,600 m), and sea level (75 m) altitudes. The authors found that Hif-1α expression were increased in women living in high altitudes. Elevated Hif-1α level induced many vascular circulating proteins, which may role in placental pathologies. Ietta et al.^32^ showed that, in human placenta, Hif-1α expression is increased when oxygen tension is low, and decreased when oxygen tension is high. They also stated that Hif-1α is localized in cytotrophoblasts in early weeks of pregnancies, and function to change expression of many genes during placental development. Another study showed that during placentogenesis constitutive expression of Hif-1α in trophoblastic cells leads to significant decrease in birth weight^33^.

In normotensive group, Hif-1α reaction was mainly negative, but it was positive in some fibroblast cells and syncytial node (Fig. 3c). In the placenta with HELLP syndrome, Hif-1α expression was positive in small decidual cell nuclei, but mainly positive in inflammatory cells in the maternal region (Fig. 3d).

## Conclusion

HELLP syndrome causes degeneration in syncytial development and decidual cells. Hypoxia causes vascular endothelial dysfunction. Therefore, Hif-1α signal and SOX9 transformation factor may play an important role in determining the severity of preeclampsia and also in the regulation of cell proliferation and angiogenesis.

## Data Availability

All data sets were generated or analyzed in the current study.
